# Incidence and patterns of surgical glove perforations: experience from Addis Ababa, Ethiopia

**DOI:** 10.1186/s12893-017-0228-8

**Published:** 2017-03-20

**Authors:** Abebe Bekele, Nardos Makonnen, Lidya Tesfaye, Mulat Taye

**Affiliations:** 1 School of Medicine, Addis Ababa University, PO BOX 3560, Addis Ababa, Ethiopia; 2 Drexel University, Philadelphia, USA

## Abstract

**Background:**

Surgical glove perforation is a common event. The operating staff is not aware of the perforation until the procedure is complete, sometimes in as high as 70% of the incidences. Data from Ethiopia indicates that the surgical workforce suffers from a very surgery related accidents, however there is paucity of data regarding surgical glove perforation.

The main objective is to describe the incidence and patterns of surgical glove perforation during surgical procedures and to compare the rates between emergency and elective surgeries at one of the main hospitals in Addis Ababa Ethiopia.

**Methods:**

This is a prospective study, performed at the Minilik II referral hospital, Addis Ababa. All surgical gloves worn during all major surgical procedures (Emergency and Elective) from June 1-July 20, 2016 were collected and used for the study. Standardised visual and hydro insufflation techniques were used to test the gloves for perforations. Parameters recorded included type of procedure performed, number of perforations, localisation of perforation and the roles of the surgical team.

**Results:**

A total of 2634 gloves were tested, 1588 from elective and 1026 from emergency procedures. The total rate of perforation in emergency procedures was 41.4%, while perforation in elective surgeries was 30.0%. A statistically significant difference (*P* < 0.05) was found in between emergency and elective surgeries.

There were a very high rate of perforations of gloves among first surgeons 40.6% and scrub nurses 38.8% during elective procedures and among first surgeons (60.14%), and second assistants (53.0%) during emergency surgeries.

Only 0.4% of inner gloves were perforated. The left hand, the left index finger and thumb were the most commonly perforated parts of the glove. Glove perforation rate was low among consultant surgeons than residents.

**Conclusions:**

Our reported perforation rate is higher than most publications, and this shows that the surgical workforce in Ethiopia is under a clear and present threat. Measures such as double gloving seems to have effectively prevented cutaneous blood exposure and thus should become a routine for all surgical procedures. Manufacturing related defects and faults in glove quality may also be contributing factors.

## Background

Since their introduction in the late 1880’s, surgical gloves have been vital in protecting the surgical team from exposure to pathogens during surgery, especially viruses such as hepatitis B, hepatitis C and the human immunodeficiency (HIV) virus [1–3]. Although surgical gloves are the main barrier between the surgeon and the patient, glove perforation is a common event and can reach 78%, especially during emergency procedures [3, 4]. Surgical gloves are important in creating a sterile environment in the operating room, therefore, perforation of gloves during surgical procedures can create a potential route for spread of pathogens. Originally the main role of surgical gloves was to limit the spread of pathogens from the surgeons’ hands into the patient by maintaining an aseptic environment and minimising the chance of surgical site infections. Gloves also provide a barrier between surgical personnel and patient. Glove perforation therefore means this barrier is compromised and this provides a potential route to the surgical personnel getting contaminated by the patients’ body fluids. In order to prevent such problems, it is necessary to understand the rate of glove perforation along with steps that alter its incidence.

It was been shown that the risk of perforations depends on the type of surgery performed, ranging from 7% in urological surgery up to 65% in cardiac surgery [5, 6]. In addition, studies that have compared the rate of perforation among elective and emergency surgeries have shown that perforation to be higher in emergency surgical procedures [7, 8]. In addition, one of the major challenges encountered during surgical glove perforation is that the operating staff is not aware of the perforation until the procedure is complete, sometimes in as high as 70% of the incidences [1].

One of the approaches suggested to be helpful in reducing the risk of infection prevention during glove perforations is the use of double gloves instead of single glove [9–11]. Previous studies have demonstrated that when double gloves are used, the inner glove had less perforations (as low as 2%), hence reducing cross-infection [9, 10].

There are few studies performed in Ethiopia regarding glove perforations, however, two studies have shown that there is a very high rate of intra-operative accidents to the operating team including glove perforation and needle sticks [11, 12]. Therefore, the main objective of this study is to describe the prevalence of surgical glove perforation during surgical procedures and to compare the rates between emergency and elective surgeries at one of the main hospitals in Addis Ababa Ethiopia. We believe this will be the first of such reports from Ethiopia and can serve as a base line publication of subsequent wider studies.

## Methods

This is a prospective study, done at the Minilik II referral hospital, Addis Ababa, Ethiopia. The Minilik hospital is an affiliate hospital of the Addis Ababa University where surgical residents and consultant surgeons are involved in the care of surgical patients on regular basis. The hospital has 110 surgical beds and 6 operating theatres and approximately 60 cases are operated every week. The study received permission and ethical clearance from the research and publication committee of the Department of Surgery, Addis Ababa University.

All Gloves worn during all major surgical procedures (Emergency and Elective) from June 1-July 20, 2016 were collected and used for the study. The gloves used by each health professional for each procedure were separately collected in a labelled collecting box provided for each surgery; members of the surgical team placed their used gloves immediately following the operation.

At the end of each procedure, data regarding the specific surgical procedure was collected by using a pre-approved structured format. During the study period, it was noted that different brands of surgical gloves from different manufactures were used and no specific selection was made among the different brands.

After each procedure, the gloves were taken from the operating room for testing. Standardised visual and hydro insufflation techniques were used to test the gloves for perforations [13, 14]. Testing and data collection was done by the authors.

1. ***Visual***: each glove was inspected visually for perforations. The examiner is blinded to the surgery type.
2. ***Hydro Insufflation***: Each glove was tested by a standardised water-leak test. Gloves are filled with 1000 ml of water and methylene blue solution. Followed by manual compression on the wrist of the glove for 1 min to reveal any holes. The leaking of blue-water would indicate perforation.

Following testing, the number of perforations in each glove, the perforated layer of glove, and the location of perforation were all recorded. The data was analysed by using chi-squared method, with a statistical significance of *p* < 0.05.

## Results

The study analysed perforation in 2634 gloves, with two sets of data being obtained for testing. The first set looked at glove perforation in 1588 (794 pairs) of gloves from elective procedures, while the second set analysed perforations in 1046 (523 pairs) of gloves obtained from emergency operations. The total rate of perforation in emergency procedures was 41.4%, while perforation in elective surgeries was 34.3% (*P* < 0.05).

### Set I

A total of 1588 gloves were collected from elective surgeries, 436 gloves were used by the first surgeons, 414 by the first assistants, 306 by the second assists, 30 by the third assistants, and 402 by scrub nurses. The results show that during elective procedures, a total of 546 (34.3%) were found perforated (See Table 1).

**Table 1:** Rate of glove perforation among the operating team during elective surgery

Role	Total gloves used (*N* = 1588)	Perforated (*N* = 546)	Non-Perforated (*N* = 1042)
Surgeon	436	176 (40.4%)	260 (59.6%)
First assistant	414	120 (28.9)	294 (71.01%)
Second assistant	306	90 (29.4%)	216 (70.6%)
Third assistant	30	4 (13.3%)	26 (86.7%)
Scrub nurse	402	156 (38.8%)	246 (61.2%)

Higher rates of perforations were seen among first surgeons 40.4% and scrub nurses 38.8% compared to first assistant (28.9%), second assist (29.4%), and third assist (13.3%). This difference was found to be statistically significant (*p* < 0.05).

**Fig. 1:**
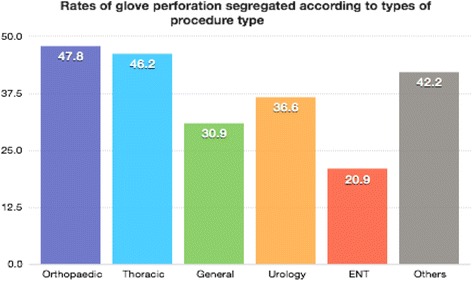
Rates of glove perforation segregated according to types of Surgical procedure type

The index and middle fingers of the left handed gloves were commonly perforated during elective procedures. Orthopaedic and Cardio-thoracic procedures were associated with significantly more perforations as compared to other procedures, occurring in 44 (47.8%) and 86 (46.2%) of the gloves respectively (See Fig. 1). Double gloves were worn in 100% of cases, however perforation of both the inner and outer gloves were detected in only 21(0.4%) of the gloves perforated during elective procedures.

### Set II

One thousand forty-six gloves were obtained for testing from emergency surgeries: 270 were used by the first surgeons, 288 by first assistants, 212 by second assistants, and 276 by scrub nurses. The results show that during emergency procedures, a total of 465 (44.4%) were found perforated (See Table 2). First surgeons, 176 (40.4%), and second assistants 90 (29.4%) had the highest rate of perforation, followed by scrub nurses 156 (38.8%), and first assistants 120 (28.9%).

**Table 2:** Rate of glove perforation among operating team during emergency surgery

Role	Total gloves used (*N* = 1046)	Perforated (*N* = 465)	Non-perforated (*N* = 581)
Surgeon	270	162 (60.14)	108 (39.86%)
First Assist	288	78 (27.2%)	210 (72.8%)
Second Assist	212	112 (53.3%)	100 (46.7%)
Scrub nurse	276	113 (41.2%)	163 (58.8%)

## Discussion

Literature review has shown that surgical gloves can be punctured in a number of ways. Most are self-inflicted and occur most commonly during wound closure [12–16]. In some cases, poor assistance by the operating team or excessive fat are listed as contributing factors [15]. In addition, major operations involving use of the mass closure technique carry a high risk of glove puncture for the operating surgeon. One previous study has shown that 12 of 21 known perforations occurred during the mass closure of surgical wound [16]. Although it is rare, the glove type, material, and brand, may also have an influence on the incidence of perforations, as latex gloves are more resistant to puncture than vinyl ones [1].

Our study has documented that the rate of glove perforation is high for both elective and emergency procedures. The overall rate of perforation observed, for both elective and emergency procedures, is 38.3%. This is higher than those reported in previous studies which range from 8.8 to 24% [17–20]. We believe the following factors may play a significant role in the increased rate of glove perforations seen in our study. Most, if not all, emergency procedures are performed by surgical residents with relatively limited surgical experience, this can potentially lead to higher rates of perforation. Additionally, during the study period, there was a serious lack of variety in glove sizes. Because there are limited options for glove sizes, a portion of the surgical team uses gloves that are either too big, or too small. The use of inappropriate sized gloves might contribute to the increased rate of glove perforations. Furthermore, developing countries like Ethiopia depend on import of cheap and lower quality products, hence manufacturing related perforations may also be more prevalent here. However, additional studies need to be conducted to confirm the role of glove quality in rates of glove perforation.

In terms of location of glove perforations, our study has shown that the left index and middle fingers are the most commonly perforated. This finding is also reflected in other studies [7, 20]. A possible explanation for this is that most perforations occur during suturing and the needle holder is often held with the right hand and the needle may accidentally perforate the glove of the opposite hand [20].

In analysing the different glove layers, the rate of perforation for the inner glove was shown to be 0.4%. Hence, double gloving appears to be a sufficient form of protection against perforations in most surgical procedures. Previous studies have shown that the use of double gloves during operations markedly reduces the risk of contamination by blood and other body fluids, compared to single gloves [10, 21–23]. The literature on double gloving is extensive, a past study has demonstrated that the inner glove perforation rates decreased from 20.8 to 2.5% for the operating team members when they double gloved [24, 23]. These results were more dramatic for the surgeon him or herself with rates decreasing from 34.7 to 3.8%. In a separate study, blood contamination of the hands decreases from 13 to 2% with the use of double gloves [22].

When looking at perforations among surgical staff, our study has found that surgeons had the highest rate of perforation for both elective and emergency procedures compared to other operating staff. Surgeons also sustained a statistically significant increased rate of glove perforation during emergency procedures than elective procedures. (60.14% vs. 40.4%, *P* < 0.01). In addition, scrub nurses sustained the second highest rate of glove perforation during elective procedures. These findings are in agreement with the literature, as various studies have shown that surgeons and scrub nurses have the highest rates of glove perforations [7, 8, 20]. One possible explanation is that compared to first, second, and third assists, the main surgeons, are the ones using instruments directly and for longer durations in procedures with increased risks for perforations.

There is also a direct exchange of sharp surgical instruments and other consumables between the surgeon and the scrub nurse, hence potentially increasing the risk perforation in both.

Overall, investigating incidents of glove perforations is an increasingly important aspect of preventing contaminations. Surgical site infections contribute significantly to post-operative morbidity and mortality all over the world [9]. In addition to putting patients at risk, glove perforations can cause potential harm for surgical personnel. Intra-operative exposure to blood and other bodily fluids has been observed in several cases due to glove perforation [11]. While surgical site infections in patients is most often bacterial, viral infections tend to affect surgical staff, the most Blood-borne infections such as Human Immunodeficiency Virus (HIV), Hepatitis B, and Hepatitis C are the leading concern for a surgical team. By utilizing improved gloving techniques, it is possible to create a safer work space.

The rate of surgical glove perforation has not been studied in Ethiopia. So far, two studies have shown than the surgical workforce in Ethiopia are working under very common and repeated intra operative risk for exposure to blood by needle sticks, cut with sharp objects and splash to the face and eyes [11, 12]. Additionally, it is also documented that most of the surgeons in Ethiopia are not fully vaccinated against hepatitis B [25]. This working environment should not be acceptable by any standards. In addition to the importance of protecting the patients and the health of surgeons, the country should also envision its economic interest in protecting its investment in the surgeons and the entire surgical workforce.

## Conclusion

Our study has shown that there is a notably high rate of surgical glove perforation in Ethiopia, especially during emergency procedures. Therefore, it may be necessary to ensure that the quality and fit of gloves that are being used for surgical procedures are examined prior to the start of all procedures. Furthermore, post procedural glove checks are recommended in case of suspected glove perforation. This will allow surgical staff to take the necessary steps to avoid contamination, and infection. The use of using different colour indicator gloves as the inner glove that allows earlier detection of a glove penetration is highly recommended so that the surgical personnel can change their gloves once they realise they are perforated [26].

Since glove perforations are common worldwide, surgeons and the surgical team are expected to adhere to the universally accepted standards of avoiding occupational injuries. In addition, Hepatitis B vaccination should be required by law for employment and during registration/accreditation. These necessary immunisations should be provided free of charge to all surgical professionals as protective measures. Moving forward, studies that focus on exposure to blood, and infectious fluids as a result of glove perforations would further enhance our understanding of surgical risks.
